# Application of Two-Stage Variable Temperature Drying in Hot Air-Drying of Paddy Rice

**DOI:** 10.3390/foods11060888

**Published:** 2022-03-21

**Authors:** Xingang Xu, Tianyuan Zhao, Jianing Ma, Qi Song, Qiao Wei, Weihong Sun

**Affiliations:** 1 College of Agricultural Engineering, Jiangsu University, Zhenjiang 212013, China; 2212016006@stmail.ujs.edu.cn (X.X.); zhaotianyuan0711@163.com (T.Z.); majianing7581@163.com (J.M.); songqi_ujs@163.com (Q.S.); 2 Jiangsu Runguo Agricultural Development Co., Ltd., Zhenjiang 212013, China; zhenzhong1250@163.com

**Keywords:** drying, paddy rice, moisture content, variable temperature

## Abstract

The aim of this study was to investigate the effect of two-stage variable temperature drying (VTD) on the quality and drying efficiency of paddy rice in the hot air-drying process. A constant temperature of 50 °C (CTD) was used as a control group. VTD and CTD methods were applied in a 15 ton batch type recirculating grain dryer. Three aspects (appearance quality, physical and chemical properties, taste quality) of the paddy rice samples from the dryer were measured and compared. It was observed that paddy rice with an initial moisture content of 25.3% (wet basis) was dried to 14% (wet basis). Compared to CTD, the VTD method could reduce the drying time and fissuring rate by 0.7 h and 42%, respectively. It had a head rice yield (HRY) of 78.45%, compared to 76.45% by CTD. The fatty acid content of the VTD samples was 2.28% lower than those of CTD, and it exhibited a 34% decrease in amylose content. These results show that two-stage VTD is an advanced hot air-drying method that can be used to improve the quality of dried paddy rice, maintain efficiency, and reduce the cost of the drying process by minimizing the rate of energy consumption.

## 1. Introduction

Paddy rice is one of the most common grain crops planted in the world, and more than half of the world’s population is fed by rice [1,2]. Paddy rice can be stored well for a long period of time if it is kept clean, dry, and away from predators. Freshly harvested paddy rice with a high initial moisture content will increase the possibility of decomposition and therefore enhance postharvest losses [3], so moisture content (MC) should be less than 14% in the case of long-term storage [4]. Hence, timely and effective paddy rice drying after harvesting has become an extremely vital procedure to the rice-producing and processing industry [5,6]. The traditional drying method used by Asian farmers is outdoor sun drying. It can be conducted directly on the farm, and the advantage of using free and renewable energy has enabled the widespread use of this conventional method by local farmers. However, outdoor sun drying can lead to deterioration in both the quality and quantity of rice due to weather changes and attacks by living organisms. Furthermore, this kind of process requires an enormous amount of labor and time. Currently, the mechanical drying method is commonly used for drying paddy rice. Mechanical drying can readily meet the requirement of large-scale production and is easier to control [7].

Paddy rice is a type of heat-sensitive grain covered by stiff and dense hulls which can protect the kernel against damage during storage and prevent the migration of moisture within the grain. Therefore, kernel breakage can easily occur during the drying process thus influencing the quality and price [2,8].

Some common drying methods include: microwave [9,10], infrared [11], vacuum [12], and hot air-drying. However, because of the high initial capital costs, none of those drying technologies are so widely used as hot-air drying in developing countries [13,14]. Presently, hot air-drying is one of the most widely used drying methods. It is characterized by low heat consumption, direct heating and drying, and uniform drying. It is mostly used in the process of mechanized drying of paddy rice. The hot-air dryer removes the moisture from within paddy rice by a hot airflow. In the hot air-drying procedure, a constant temperature of 50 °C is used. This leads to the production of rice with a high fissuring rate, since the fissured rice is easily broken during milling; the head rice yield is decreased (HRY) [15]. The HRY is an indispensable index to evaluate the drying method of paddy rice [16]. To reduce this occurrence, this study adopted a two-stage variable temperature drying process using varied drying temperatures for different moisture contents to reduce post-harvest losses. This method with optimal quality and minimum energy consumed can maintain breakage losses down at a lower level.

This study aims to develop a viable method for the hot air-drying of grains, with the following specific objectives: (1) to obtain the optimal two-stage VTD method in the laboratory; (2) to apply the optimal two-stage VTD method in the 15 ton batch type recirculating grain dryer; (3) to compare VTD with CTD method with respect to the quality and drying efficiency of paddy rice.

## 2. Materials and Methods

### 2.1. Materials

Paddy rice variety Nanjing 46 (*Oryza sativa* L.) was sampled at Jiangsu Runguo Agricultural Development Co., Ltd., Zhenjiang City, Jiangsu Province, China, during the harvest season in November 2020. The fresh paddy rice was taken to the laboratory within 24 h and immediately sealed and stored in a refrigerator at 4 °C. After drying, the paddy rice was placed in a woven bag at room temperature for 7 days and then appearance quality, physical and chemical properties, taste quality of the paddy rice were measured.

### 2.2. Thin-Layer Drying of Paddy Rice

Single-factor experiments and response surface methodology (RSM) were carried out using an electric heating constant temperature blast drying oven (DHG-9003, Shanghai Jing Hong Laboratory Instrument Co., Ltd., Shanghai, China). The independent variables included temperature of first stage, temperature of second stage, and drying time (Table 1). Because the length of the drying section and tempering section of the 15 ton batch type recirculating grain dryer is 1:5, the tempering time varies with the drying time [16]. Single-factor experiments were used to determine the temperature interval and time interval that needed to be optimized. Before the drying test, the paddy rice samples were sieved to remove impurities and manually selected to remove the particles that did not meet the test requirements. When the air volume and temperature in the drying box were stable, 100.00 ± 0.2 g of rice samples was weighed and spread on an 18-mesh standard sieve to a thickness of 10 mm. They were then placed in a drying box while the time was recorded. The average ambient temperature was 18 °C and relative humidity was 55%. Paddy rice was dried and tempered according to the set time, and the samples were stored in the tempering step in a sealed bag. The tempering time and the sample mass at the end of the tempering were recorded. The drying experiment was stopped when the MC of the rice decreased to 14%.

### 2.3. Drying of Paddy Rice in Grain Dryer

**Figure 1:**
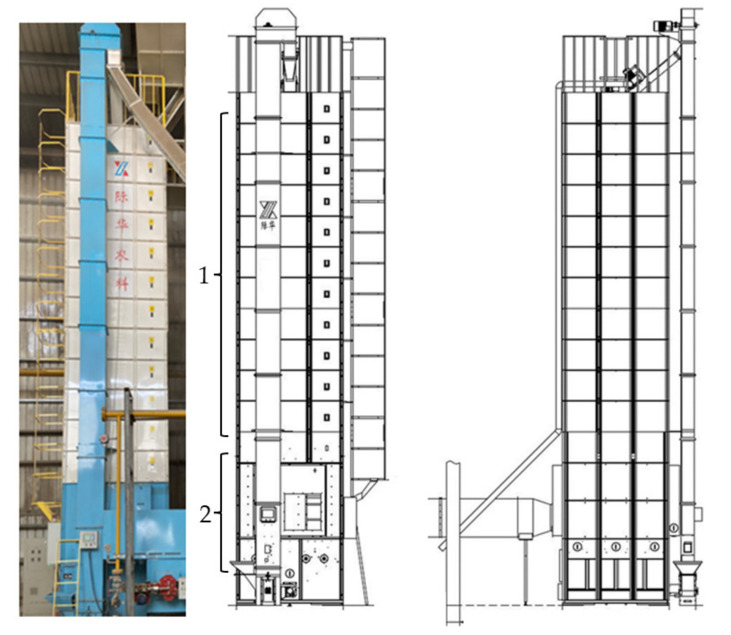
The scheme of the 15 ton batch type recirculating grain dryer: (1) the tempering section; (2) the drying section. Subfigures come from 5HXJ grain dryer instructions.

The optimal drying method obtained by response surface methodology (RSM) was carried out and applied on the 15 ton batch type recirculating grain dryer (5HXJ, Jihua Agricultural Science and Technology Development Co., Ltd., Wuhu, China). Figure 1 is the illustration of the 15 ton batch recirculating grain dryer. The dimensions of the 15 ton batch type recirculating grain dryer are shown in Table 2. It was compared with the paddy dried at a constant temperature of 50 °C, and the real-time moisture content on the grain dryer was recorded. When the MC of the rice dropped to 14.5%, a sample of the rice was collected and placed in a sealed plastic bag at 4 °C for 14 days before quality analysis. Comparisons were made from three aspects: (1) appearance quality, (2) physical and chemical properties, (3) taste quality.

### 2.4. Determination Appearance Quality of Samples of the in 15 Ton Batch Type Recirculating Grain Dryer

#### 2.4.1. The Fissuring Rate of the Samples

The samples were hulled by hand, and the cracks of 100 grains of brown rice were observed under a spotlight. The number of grains of brown rice with cracks was the crack rate of the rice. Each experiment was repeated three times.

#### 2.4.2. The Husked Rice Yield of the Samples

The Chinese National Standard GB/T 5495-2008 was adopted to determine the husked rice yield (*HURY*) from paddy rice. Briefly, 22 g ± 0.01 g of samples from paddy rice was weighed, and the immature paddy rice was picked out, weighed, and recorded as *M1*. A rice sheller (JLGJ4.5, Taizhou Grain Instrument Factory, Taizhou, China) was used to remove the husk of samples. Brown rice was weighed and recorded as *M2*. A broken rice separator machine (FOS-13X20, Zhejiang Puyun Precision Instrument Co., Ltd., Taizhou, China) was used to separate the broken rice from the samples, and the broken rice was weighed and recorded as *M3*. The *HURY* was selected according to Formula (1).

(1) $$HURY=\frac{\left(M1+M2\right)-\left(M1+M3\right)÷2}{22}\times 100$$

#### 2.4.3. The Head Rice Yield of the Samples

Inspections of *HRY* adopted the Chinese National Standard GB/T 21719-2008. The obtained brown rice (2.4.2) was placed in an adjusted rice mill (JNMJ3, Taizhou Grain Instrument Factory, Taizhou, China). The mill time was set 40 s and a broken rice separator machine (FOS-13X20, Zhejiang Puyun Precision Instrument Co., Ltd., Taizhou, China) was used to separate the broken rice from the samples. Then, the head rice was weighed and recorded as *M4*. The *HRY* was selected according to Formula (2).

(2) $$HRY=\frac{M4}{22}\times 100$$

#### 2.4.4. Color Difference

A spectrophotometer (UltraScan VIS, HunterLab, Reston, VA, USA) was used to measure the color difference of the dried paddy rice. The *L* value (lightness), *b* value (yellow/blue), and *a* value (green/red) of the dried paddy rice were measured by using the spectrophotometer [17,18]. The dried samples were shelled into brown rice, which was then crushed and passed through a 100-mesh sieve to measure the color difference. The untreated fresh brown rice was considered as, the standard rice that has not been hot-air dried at the end of the harvest. The color difference Δ*E* was selected according to Formula (3) [19,20].

(3) $$\Delta E=\sqrt{\Delta L^{2}+\Delta a^{2}+\Delta b^{2}}$$

### 2.5. Determination of Chemical Indicators

#### 2.5.1. Amylose Content

Each treatment’s paddy rice sample (0.1 g) was milled in liquid nitrogen, then combined with 1.0 mL extraction media. Amylose was measured using an amylose content assay kit (Beijing Solarbio Science & Technology Co., Ltd., Beijing, China).

#### 2.5.2. Determination of Fat Acidity

To determine fat acidity, according to the Chinese National Standard GB/T 15684-2015, the rice flour that passed through a 100-mesh sieve was used. Extracted lipid with ethanol and titrated with KOH. The indicator was phenolphthalein and the fat acidity content was measured [18].

#### 2.5.3. Crude Fat Content

Determination of crude fat content from paddy rice adopted the Chinese National Standard GB/T 5009.6-2016. Then, 2 g ± 0.001 g of processed rice flour was weighed and placed in a cylindrical filter paper. A Soxhlet extractor (SOX406, Shandong Hanon Scientific Instrument Co., Ltd., Dezhou, China), was used to determine the crude fat content of paddy rice.

### 2.6. Pasting Property of Rice

Inspections of pasting property of rice adopted the Chinese National Standard GB/T 24852-2010. Using a rapid visco-analyzer, the pasting characteristics of rice flour were determined (RVA-TecMaster, Perten Co., Ltd., Stockholm, Sweden). Rice flour (3.0 g, weight of standard sample; 12%, wet basis) and distillated water (25.0 g, weight of standard water) were heated to 95 °C at a rate of 12 °C/min after being kept at 50 °C for 1 min. The rice paste was kept at 95 °C for 2.5 min, then cooled down to 50 °C at a rate of 12 °C/min. The paste was then kept at 50 °C for 1.4 min [21]. Viscograms were used to determine the peak, hold, and final viscosity values.

### 2.7. Extraction of Starch and Observation of Granular Morphology

The separation of starch adopted the alkaline extraction method [22]. According to the method of Dawei Zhu et al. [21], starch was extracted from rice flour which passed through a 100-mesh sieve. Fifty grams of rice flour and 400 mL of 0.2% NaOH solution were added to a beaker which was placed at room temperature for 24 h. Then, a 200-mesh sieve was used to filter the homogenate. After centrifugation, the bottom white layer of the filtrate was washed with distilled water. Lastly, the starch was dried with a drying oven (DHG-9003, Shanghai Jing Hong Laboratory Instrument Co., Ltd., Shanghai, China) at 40 °C then passed through a 200-mesh sieve. Starch samples were coated with gold, used a thermal field emission scanning electron microscope (JSM-7001F, JEOL, Tokyo, Japan) to observe [23].

### 2.8. Sensory Assessment of Cooked Rice

For the sensory analysis of cooked rice, ten trained panelists were chosen. Taste, appearance, softness, flavor, and cold rice texture were all assessed as sensory parameters. The sensory evaluation of cooked rice was carried out according to the Chinese National Standard GB/T 15682-2008. Each sample was made using 100 g rice. The rice was weighed and washed three times with distilled water, and the washing time was controlled within 3–5 min. Then, 1.1 sample weight of water were added to each sample. The samples were immersed for 30 min at 25 °C then steamed for 40 min in a steamer and simmered for another 20 min. Sampling glasses were filled with various samples. Panelists tasted and rated the samples in a timely manner. The highest score was 25 for taste, 30 for softness, 20 for flavor, 20 for appearance, and 5 for cold rice texture. It was the standard for scoring cooked rice [18,24].

### 2.9. Texture Profile Analysis (TPA) of Cooked Rice

Three samples of cooked rice grain were chosen and placed on the bottom plate. A TA.XT-Plus Texture analyzer (Stable Micro Systems Ltd., Surrey, UK) with a P/36R cylindrical probe attachment was used for measurements. The probe descended at 1 mm/s, returned, and the compression cycle was repeated. The compression was set to 70% strain, a time of 2 s, and a trigger force of 5 g. Texture measurements were taken five times for each cooking duplicate [18].

### 2.10. Statistical Analysis

The data were analyzed using analysis of variance and Duncan’s multiple range test and the independent sample *t*-test with SPSS 22.0 software and Excel 2021. The Design Expert 10.0 software was used to design the RSM and analyze the data.

## 3. Results

### 3.1. Single Factor Experiments Results and RSM Design and RSM Results

**Figure 2:**
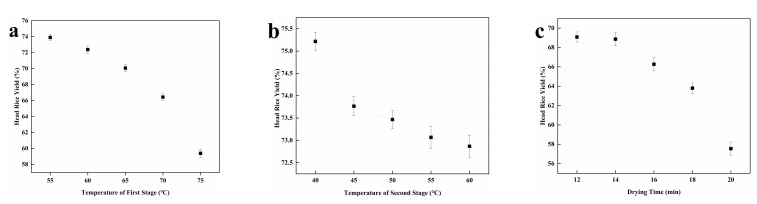
The single factor experiments results. (**a**) the head rice yield of the paddy rice in the first stage, the moisture content of rice samples was 23% ± 0.1%, (**b**) the head rice yield of the paddy rice in the second stage, the moisture content of rice samples was 18% ± 0.1%, (**c**) the head rice yield of the paddy rice at different drying time.

As shown in Figure 2, the HRY of paddy rice decreased with the extension of drying time and the increase in drying temperature. In the first stage, the drying temperature has a great influence on the HRY of the rice. The interval that needed to be optimized is shown in Table 3. The RSM was carried out with the HRY of paddy rice and total drying time as the response. From the equation in Table 4, the optimal two-stage variable temperature drying method is as follows: 60 °C in the first stage, 45 °C in the second stage, and a drying time of 12 min.

### 3.2. Applied on the 15 Ton Batch Type Recirculating Grain Dryer

The optimal two-stage variable temperature drying method obtained from RSM was applied to the 15 ton batch type recirculating grain dryer, in which the drying is divided into two stages. The initial drying temperature in the first stage was 60 °C. The second stage of drying, which started when the MC of the paddy rice was reduced to 17–18%, reduced the drying temperature to 45 °C. Hot-air drying included both heat and mass transfer processes, combined with the particularity of rice drying. The drying temperature is the key factor in the entire drying procedure. The initial MC of the freshly harvested paddy rice was above 23% and the rice was dried until the moisture content ended at 14%. The cycle speed of the dryer was also adjusted to two cycle speeds: 30 Hz and 50 Hz. Table 5 shows the comparison between the two drying methods of two-stage VTD and 50 °C CTD. As shown in Table 5, compared to CTD, VTD uses less time to dry the paddy rice. This is because the 60 °C used in the first stage of the VTD improves the drying speed, thus reducing the consumption of resources while ensuring drying efficiency.

### 3.3. Appearance Quality

**Figure 3:**
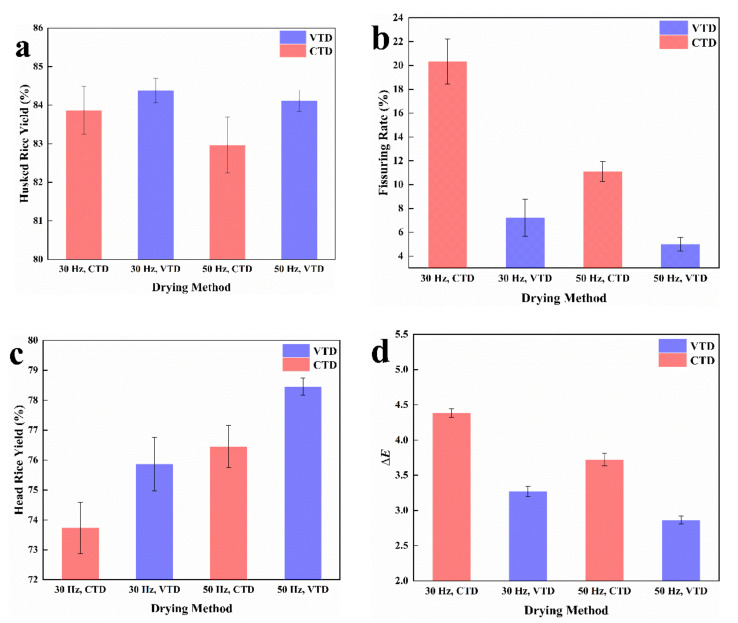
Quality indicators for the appearance of rice under different drying methods: (**a**) the husked rice yield of the paddy rice dried at VTD and CTD; (**b**) the fissuring rate of the paddy rice dried at VTD and CTD; (**c**) the head rice yield of the paddy rice dried at VTD and CTD; (**d**) total color difference value (Δ*E*) of the paddy rice dried at VTD and CTD.

The appearance quality of paddy rice using the two different drying methods was measured and it was found that the husked rice yield and fissuring rate of the paddy rice dried at VTD were significantly lower than those dried at CTD. As shown in Figure 3, the HRY of 50 Hz VTD was greater than those of 30 Hz CTD and 50 Hz VTD. This indicated that the key drying temperature affected the quality of the paddy rice when the moisture content of the paddy rice was below 18%. There existed a limit for the percentage points of moisture content removal per drying pass beyond which HRY would decrease [25]. It was observed that when the paddy rice changes from a rubbery state to a glassy state, the temperature at this point was 5 °C lower for the two-stage VTD method than for the CTD method. The high quality of the paddy rice was guaranteed. However, 30 Hz VTD had a lower HRY than 50 Hz CTD. The rest of the indicator comparisons are for paddy rice dried at 50 Hz. The confrontation of the moisture gradient with the temperature gradient of moisture movement can be extremely strong when the drying temperature is high and finally leads to serious fissuring. Numerous studies have shown that the reaction of the fissure ratio to drying temperature is consistent—markedly enhanced along with the increase in drying temperature [26,27,28]. The initial water content of paddy varies from place to place. The 30 Hz CTD and 30 Hz VTD experimental rice samples were high moisture paddy rice, so the fissure ratio was high. The traditional theory considered that there existed an inner stress gradient [29]. Thus, when rice absorbs moisture or is being heated, a crack will occur if the inner stress is greater than the tensile strength, and particles will break during the hulling and milling process; therefore, high-temperature drying was not suggested. However, since research has shown that material properties of rice kernels [30], which are affected by MC and drying temperature, play a vital role in rice drying, the glass transition temperature concept has been used to explain trends in fissure formation [31].

The color difference of each processed sample was different and the total color difference value (Δ*E*) of VTD was lower than CTD. The temperature in the second stage may have an impact on the degree of color difference in the VTD. The color of paddy rice was influenced significantly by the drying procedures [32].

### 3.4. Chemical Indicators

As shown in Table 6, the drying temperature had almost no effect on the crude fat of the paddy rice. However, the fatty acid and amylose content of rice dried at CTD was higher than those dried at VTD, and the difference in amylose was more obvious. Rice quality was highly linked to amylose content [33,34]. The amount of amylose in rice has a direct impact on water absorption throughout the cooking process [35,36]. Rice with a high amylose level has a hard texture, low viscosity, dark color, and the rice grains are dry and fluffy with no scent under normal conditions, whereas rice with a low amylose content has a soft texture, high viscosity, and poor elasticity [35,36]. Research has shown that rice with a higher amylose content and lower protein content tends to have a better aroma and flavor [37,38]. Inappropriate drying conditions will alter some intrinsic physicochemical characteristics of rice, thus destroying the expected aroma and flavor. This possibility can even be increased if paddy rice is dried at high temperatures for a certain duration of time [39].

### 3.5. Microstructural Observation of Starch Granules

**Figure 4:**
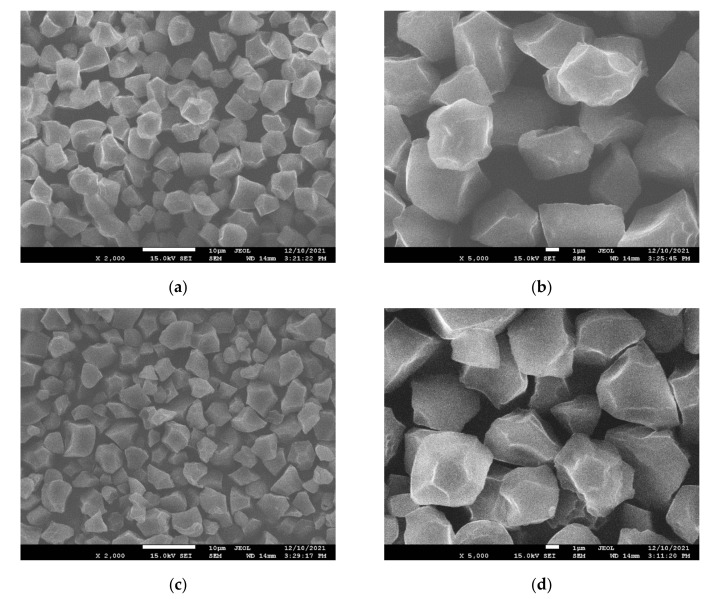
SEM of starch extracted from paddy rice under 50 Hz CTD and 50 Hz VTD: (**a**,**b**) 50 Hz CTD; (**c**,**d**) 50 Hz VTD.

Rice starch, as the most significant nutrient in rice, plays a critical role in the grain’s quality. The structure and properties of starch are the main factors affecting the physicochemical properties and palatability of rice [40]. The extraction of starch from rice samples was performed using two different drying methods: 50 Hz CTD and 50 Hz VTD. SEM photos of starch granules revealed an uneven surface with angular and polygonal features (Figure 4). Regardless of their amylose level, the native starches exhibited comparable morphological properties. Native starches were structurally homogeneous and polygonal in shape. This is in line with Indira Govindaraju’s research [23]. It shows that the 60 °C used in the first stage of the VTD method does not significantly affect the rice grain [21,23,39,40].

### 3.6. Pasting Properties of Rice Samples

**Figure 5:**
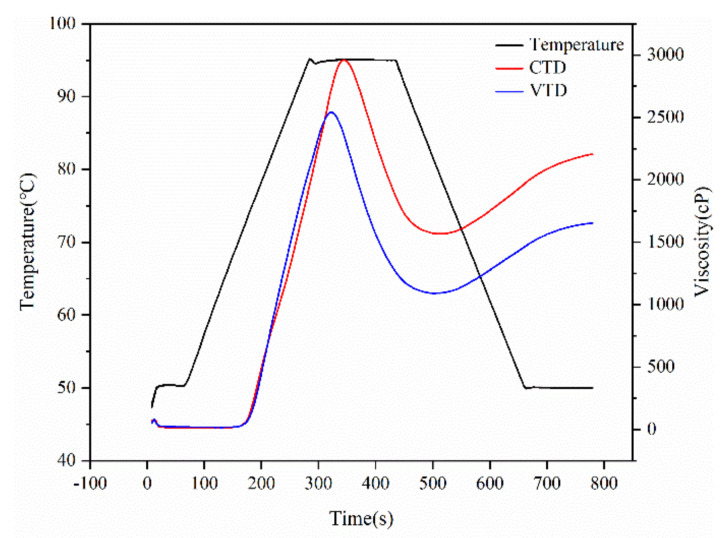
RVA profiles of two samples.

Figure 5 and Table 7 depict the rapid visco-analyzer (RVA) profiles of rice flour samples, as well as data such as the peak viscosity (PV), breakdown, final viscosity (FV), and pasting temperature (PT) [24]. It has been shown that higher proportions of amylopectin short chains and smaller proportions of amylopectin long chains may result in higher peak viscosity and breakdown values, as well as a softer and stickier texture in cooked rice [38]. Rice hardness has a highly significant positive association with setback values and a highly significant negative correlation with breakdown values, indicating that the RVA profile of paddy rice is closely related to its eating quality. This is why VTD rice tastes better than CTD rice, which is intrinsically associated with the usage of 45 °C in the VTD process.

### 3.7. Cooked Rice’s Sensory Characteristics

**Figure 6:**
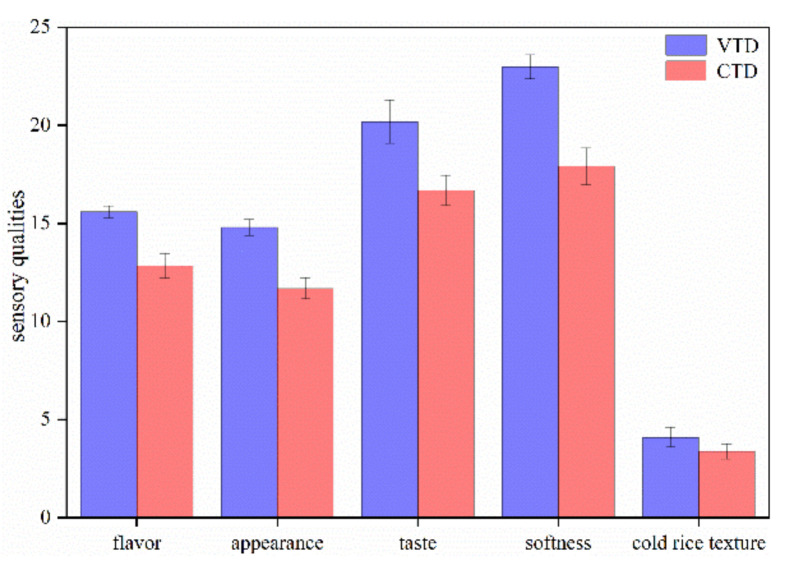
The sensory qualities of cooked rice.

Unlike most food crops, rice is generally eaten whole without seasoning, making the sensory properties of the rice itself significant. A small variation in sensory properties, especially aroma, can make rice highly accepted or totally unacceptable to consumers [24]. Consequently, aroma and flavor have been rated as the major criteria to describe the ultimate acceptance of rice by consumers [41]. Figure 6 shows the sensory qualities of cooked CTD and VTD rice. VTD rice received higher sensory ratings than CTD rice. It had a stronger aroma, suitable viscosity, and firmness than CTD rice. The VTD rice had a better appearance than CTD. One study showed that the drying temperature has a negative effect on the sensory characteristics of paddy rice [42]. The drying temperature impacts the texture of the rice in the second stage of VTD. At this time, the drying temperature of CTD was 5 °C higher than VTD. The quality and taste of VTD rice were generally superior to those of CTD rice. Therefore, VTD rice will be more favored by consumers in the market.

### 3.8. Changes in the Textural Features of Paddy Rice

The textural qualities of rice grains were assessed using rice from the taste rating experiment, which had been left at room temperature for an hour before being measured. The effects of different drying processes on the texture of rice are presented in Table 8. In comparison to VTD, CTD increased hardness, adhesiveness, gumminess, and chewiness. Apart from this, CTD decreased springiness. Among them, the effect of VTD on the hardness of paddy was more obvious. The most influential is the second stage of the drying process. According to a previous study, this is closely related to the variation in amylose content in rice [43]. The increase in insoluble amylose content was positively correlated with rice hardness and negatively correlated with the springiness of rice [44]. Studies have shown that the hardness of rice is related to the size of the amylose molecule and the ratio of long amylose chains [45]. It was clear that the first stage of the VTD method had little effect on the paddy rice. Texture qualities were inseparable from the sensory characteristics of cooked rice. At the same time, texture qualities also provided a powerful explanation for the evaluation of rice taste quality.

## 4. Discussion

Hot air-drying includes both heat and mass transfer processes. Combined with the particularity of rice drying, the drying temperature is the key factor in the entire drying procedure [14]. Paddy rice is commonly dried and stored with its husk; within the siliceous husk lies the brown rice kernel, composed of several layers of fibrous bran firmly attached to the core kernel which contains a large proportion of starch and some protein [37]. Characteristics shown in the processes of paddy hulling and milling are known as milling quality, including husked rice yield, fissuring rate, and head rice yield (HRY) [27,28]. According to the Chinese National Standard GB/T 21719-2008, HRY is defined as the weight percentage of rough rice that remains as head rice (kernels that are at least three-fourths of the original kernel length) after complete milling, which is one of the most important indicators. Fissuring or cracking can occur in a kernel during drying and will reduce HRY. However, only a serious crack can evidently lower HRY, so the fissure ratio and HRY should be combined to evaluate the rice quality [15].

The confrontation of the moisture gradient with the temperature gradient of moisture movement can be extremely strong when the drying temperature is overtopped and finally leads to serious fissuring. The theory of glass transition points out that if the drying temperature is lower than the glass transition temperature of grain, the formation of fissuring can be prevented [31,41]. This makes the determination of drying temperature more concise.

By concluding the research on physical and chemical composition changes of rice after drying experiment, a conclusion has been drawn—high drying temperature is the main factor resulting in a decline in aroma and flavor, as differences in amylose content were found in the rice after drying, and that the critical drying temperature of rice with a higher initial moisture content should be lower [42,46]. An inappropriate drying temperature has a negative effect on energy consumption as well. For the initial moisture content of less than 23%, a drying temperature of under 39 °C was suggested to ensure good drying quality and energy utilization [47]. However, in order to effectively balance the drying efficiency with the quality of rice and energy consumption, in the second stage of drying, 45 °C was selected on the 15 ton batch type recirculating grain dryer. With the development of drying technology, an increasing number of studies have been conducted to explore the relationship between the drying conditions and the post-drying quality of rice. Requests of paddy rice drying have been transferred from a single high productivity to a high HRY, and then to the cooking and eating quality.

## 5. Conclusions

In this study, a two-stage VTD method was used in the 15 ton batch type recirculating grain dryer. Different drying temperatures were adopted for different moisture contents of paddy rice, and 18% moisture content of the rice was used as the time point for a change in temperature. The effects of CTD and VTD on the drying characteristics and physicochemical parameters of paddy rice were compared. The paddy rice from the VTD method had a 2% higher HRY and a 42% lower fissuring rate, reducing the drying time by 5% compared to CTD, improving the drying efficiency, and reducing energy consumption. It was also shown that 60 °C used in the first stage of the VTD had no significant impact on the quality, as the process was solely based on the reduction in free water in the rice. The amylose content was reduced by 34% and the fatty acid content by 2.3% compared to CTD. The paddy rice of VTD scored higher in terms of taste quality and was more popular than those from CTD.

This paper is only limited to the paddy rice variety Nanjing 46, and the effect of this drying method on other rice varieties needs to be explored. However, to meet the requirements of the international market, it needs further research and improvement. This study shows that VTD, as an efficient drying method, can be applied to improve the hot air-drying process and to produce paddy rice with high quality.

## Figures and Tables

**Table 1:** Independent variables and actual levels used in single factor experiment design.

Level	Temperature of First Stage (°C)	Temperature of Second Stage (°C)	Drying Time (min)	Tempering Time (min)
1	75	40	12	60
2	70	45	14	70
3	65	50	16	80
4	60	55	18	90
5	55	60	20	100

**Table 2:** The dimensions of the 15 ton batch type recirculating grain dryer.

Model	Weight of Dryer (kg)	Length, Width and Height (mm)	The Power of the Dryer (kw)	Capacity(kg)
5HXJ-15	3420	3575 × 2990 × 11,595	9.04	Paddy rice: 2800–15,000
				Wheat: 3400–18,200

**Table 3:** Factors and their coded and actual levels used in response surface methodology.

Level	A, Temperature of First Stage (°C)	B, Temperature of Second Stage (°C)	C, Drying Time (min)
1	65	50	12
0	60	45	14
−1	55	40	16

**Table 4:** The results of response surface methodology.

Response	Equation from the RSM	*p*-Value	R-Squared	Adj R-Squared	Pred R-Squared
*HRY*	= 70 − 2.19A − 1.7B − 1.22C − 1.05AB − 1.16AC − 0.1BC	<0.0001	0.9623	0.9367	0.8971
Total Drying Time	= 11.58 − 0.91A − 1.96B − 0.4C + 0.2AB + 0.025AC + 0.13BC + 0.73A^2^ − 0.12B^2^ + 1.11C^2^	<0.0001	0.9856	0.9670	0.7898

**Table 5:** Comparison of four hot air-drying methods.

Drying Method	Drying Temperature (°C)	Cycle Speed (Hz)	Initial Moisture Content (%)	Final Moisture Content (%)	Drying Time (h)
CTD	50	50	25 ± 0.3	14.0 ± 0.5	14.2 ± 0.2 ^a^
		30	29 ± 0.5	14.0 ± 0.5	14.7 ± 0.4 ^a^
VTD	60, 45	50	25 ± 0.3	14.0 ± 0.5	13.5 ± 0.2 ^b^
		30	29 ± 0.5	14.0 ± 0.5	14.5 ± 0.2 ^a^

Values expressed as the mean ± standard error (*n* = 3). According to Duncan’s multiple range test, different superscript letters in the last column are significantly different (*p* < 0.05).

**Table 6:** Chemical indicators of the paddy rice.

Test	Crude Fat (%)	Fat Acidity (mg KOH/100 g)	Amylose Content (mg/g)
VTD	1.67 ± 0.21	16.73 ± 0.52	12.35 ± 0.13 **
CTD	1.66 ± 0.31	17.12 ± 0.35	18.67 ± 0.06

Values expressed as the mean ± standard error (*n* = 3). According to the independent sample *t*-test, ** indicated groups within one column differ significantly at *p* < 0.01.

**Table 7:** Comparison of the RVA profiles.

Test	Peak Viscosity	Minimum Viscosity	Breakdown	Final Viscosity	Setback	Peak Time	Pasting Temperature
VTD	2550 ± 82 **	1091 ± 24 **	1459 ± 74	1653 ± 27 **	562 ± 14 **	5.37 ± 0.08 **	72.6 ± 0.9
CTD	2971 ± 83	1567 ± 78	1404 ± 79	2203 ± 78	636 ± 6	5.75 ± 0.10	71.9 ± 0.4

Values expressed as the mean ± standard error (*n* = 5). According to the independent sample *t*-test, ** indicated groups within one column differ significantly at *p* < 0.01.

**Table 8:** Textural features of cooked rice.

Drying Method	Hardness (g)	Adhesiveness (g s)	Springiness (%)	Gumminess (g)	Chewiness (g)
CTD	1929.9 ± 78.5	−203.2 ± 102.3	88.9 ± 3.3	916.5 ± 105.7	812.9 ± 69.8
VTD	1619.9 ± 71.3 **	−291.3 ± 107.7	89.9 ± 0.5	853.9 ± 40.0	767.3 ± 39.3

Values expressed as the mean ± standard error (*n* = 5). According to the independent sample *t* test, ** indicated groups within one column differ significantly at *p* < 0.01.

## Data Availability

The data showed in this study are contained within the article.
